# Implications of Dysnatremia and Endocrine Disturbances in COVID-19 Patients

**DOI:** 10.3390/ijms25189856

**Published:** 2024-09-12

**Authors:** Mihaela Zlosa, Barbara Grubišić, Luka Švitek, Dario Sabadi, Silvija Canecki-Varžić, Ivica Mihaljević, Ines Bilić-Ćurčić, Tomislav Kizivat

**Affiliations:** 1 Clinic for Infectious Diseases, University Hospital Centre Osijek, 4 Josip Huttler Street, HR-31000 Osijek, Croatia; mihaelazlosa@gmail.com (M.Z.); barbara.grubisic@kbco.hr (B.G.); dariocroatia@gmail.com (D.S.); 2 Faculty of Medicine Osijek, J. J. Strossmayer University of Osijek, 4 Josip Huttler Street, HR-31000 Osijek, Croatia; 3 Department of Infectology and Dermatovenerology, Faculty of Medicine Osijek, J. J. Strossmayer University of Osijek, 4 Josip Huttler Street, HR-31000 Osijek, Croatia; 4 Faculty of Dental Medicine and Health Osijek, J. J. Strossmayer University of Osijek, 21 Crkvena Street, HR-31000 Osijek, Croatia; 5 Department of Endocrinology, Internal Medicine Clinic, University Hospital Centre Osijek, 4 Josip Huttler Street, HR-31000 Osijek, Croatia; canecki.silvija@kbco.hr; 6 Department of Pathophysiology, Faculty of Medicine Osijek, J. J. Strossmayer University of Osijek, 4 Josip Huttler Street, HR-31000 Osijek, Croatia; 7 Clinical Institute of Nuclear Medicine and Radiation Protection, University Hospital Centre Osijek, 4 Josip Huttler Street, HR-31000 Osijek, Croatia; ivanm2712@gmail.com (I.M.); tkizivat@mefos.hr (T.K.); 8 Department for Nuclear Medicine and Oncology, Faculty of Medicine, J. J. Strossmayer University of Osijek, 4 Josip Huttler Street, HR-31000 Osijek, Croatia; 9 Academy of Medical Sciences of Croatia, 15 Kaptol Street, HR-10000 Zagreb, Croatia; 10 Department of Pharmacology, Faculty of Medicine Osijek, J. J. Strossmayer University of Osijek, 4 Josip Huttler Street, HR-31000 Osijek, Croatia

**Keywords:** COVID-19, sodium imbalance, inappropriate ADH syndrome, pituitary gland, adrenal gland, thyroid gland, prognosis

## Abstract

Sodium imbalance is a common electrolyte disturbance in COVID-19, often linked to disruptions in hormonal regulation. This review explores the relationship between sodium dysregulation and endocrine disturbances, particularly focusing on primary and secondary hypothyroidism, hypocortisolism, and the renin–angiotensin–aldosterone system (RAAS). Hypocortisolism in COVID-19, due to adrenal insufficiency or secondary to pituitary dysfunction, can lead to hyponatremia through inadequate cortisol levels, which impair renal free water excretion and enhance antidiuretic hormone (ADH) secretion. Similarly, hypothyroidism is associated with decreased renal blood flow and the glomerular filtration rate (GFR), which also increases ADH activity, leading to water retention and dilutional hyponatremia. Furthermore, COVID-19 can disrupt RAAS (primarily through its interaction with the angiotensin-converting enzyme 2 (ACE2) receptor), diminishing aldosterone secretion and further contributing to sodium loss and hyponatremia. These hormonal disruptions suggest that sodium imbalance in COVID-19 is multifactorial and warrants further investigation into the complex interplay between COVID-19, endocrine function, and sodium homeostasis. Future research should focus on understanding these mechanisms to develop management algorithms that address both sodium imbalance and underlying hormonal disturbances in order to improve prognosis and outcomes in COVID-19 patients.

## 1. Introduction

After the first occurrences of this respiratory viral illness in Wuhan, Hubei Province, China, towards the end of December 2019, severe acute respiratory syndrome coronavirus 2 virus (SARS-CoV-2) rapidly disseminated on a worldwide scale [1]. Despite the official announcement by the World Health Organization (WHO) that the SARS-CoV-2 pandemic has ended, coronavirus disease 2019 (COVID-19) remains a significant worldwide concern [2].

SARS-CoV-2 is a positive-sense, single-stranded RNA virus classified within the category of betacoronaviruses (betaCoV). SARS-CoV-2 is grouped within the same subgenus as the severe acute respiratory syndrome coronavirus (SARS-CoV) and the Middle East Respiratory Syndrome Coronavirus (MERS-CoV). One as well as the other have been associated with epidemics previously [3]. It has been hypothesized that SARS-CoV-2 passed from bats to intermediary hosts like pangolins and minks before spreading to humans [4].

During this global pandemic, several versions of the SARS-CoV-2 virus have been identified, with only a limited number being classified as variants of concern (VOCs) [5]. VOCs identified globally include Alpha, Beta, Gamma, Delta, and Omicron. The VOCs have increased transmissibility, enhanced the severeness of symptoms, immunological escape, and the potential to lessen the efficacy of treatments or vaccines as opposed to the original strain of COVID-19 [6]. As per the latest epidemiological update from the WHO, there are currently no SARS-CoV-2 variants meeting the VOC standard [7].

COVID-19 exhibits notable diversity in both clinical manifestation and pathophysiological characteristics, currently leading to the majority of individuals experiencing only mild or moderate symptoms [8]. Rough calculations indicate that approximately 2% of the population are asymptomatic conveyors of coronaviruses [9].

Given the dynamic nature of COVID-19, it is clear that the virus has endured numerous changes over time. Hence, the symptoms associated with this disease have also displayed a range of variations [10]. The most common are six symptoms: fever, cough or chest tightness, sputum and coryza, malaise, and fatigue [11]. Alongside examining the differences in symptom dispersal between severe and mild cases, the large meta-analysis conducted in China in 2021 revealed that certain initial symptoms such as dyspnea, pyrexia, lethargy, hemoptysis and diarrhea were more frequent in severe cases compared to mild COVID-19 cases [12].

Plenty of complications are associated with COVID-19, including pulmonary embolism, arrhythmias, cardiomyopathy, multiorgan failure, and numerous other clinical presentations [13].

Common laboratory findings encompass leukopenia (lymphopenia), increased C-reactive protein (CRP), lactate dehydrogenase and elevated transaminases. Although lymphocyte, D-dimer, and CRP levels do not possess diagnostic value, they do offer valuable information regarding the severity of COVID-19 [14].

Hyperglycemia is observed in 20–50% of hospitalized COVID-19 patients, with higher rates in severe cases [15]. The impact of COVID-19 has been shown to intensify hyperglycemia, even in those without a history of diabetes [16,17]. This hyperglycemic response is partially influenced by the stress-induced release of cortisol and the hyperproduction of cytokines which is known to enhance gluconeogenesis and insulin resistance.

The occurrence of electrolyte imbalances, including conditions like hyponatremia, hypernatremia, hypokalemia, and hyperkalemia, is prevalent among COVID-19 patients and can markedly affect the overall prognosis of the disease [18].

Sodium is a crucial electrolyte that is indispensable for various physiological mechanisms, including osmotic regulation, nerve impulse transmission, and the maintenance of fluid equilibrium. The homeostasis of sodium is sustained through complex interactions between the kidneys and multiple hormones (aldosterone, antidiuretic hormone (ADH), atrial natriuretic peptide (ANP), and brain natriuretic peptide (BNP)) that regulate sodium reabsorption and excretion. Dysregulation of these hormonal pathways can result in dysnatremia [19].

The incidence of hyponatremia among hospitalized patients ranges from 20% to 30%, with severe cases exhibiting rates that can reach 50% [20]. Hypokalemia is also a notable issue, affecting between 10% and 20% of patients, with critical cases showing prevalence rates as high as 40% [21]. Conversely, hypernatremia and hyperkalemia are less frequently encountered, affecting 2% to 15% of patients, particularly those in intensive care settings [22]. A variety of factors can lead to hyponatremia, including SIADH, the disruption of the HPT and HPA axis, primary hypothyroidism and adrenal insufficiency, a disruption in the RAAS, dehydration, pulmonary disease and baroreceptor activation, and specific pharmacological treatments. Furthermore, the inflammatory response triggered by COVID-19 may also contribute to this condition [23].

The use of glucocorticoids, notably dexamethasone, has established itself as a pivotal strategy in the therapeutic approach to COVID-19, especially among patients suffering from severe and critical conditions [24]. The use of dexamethasone is recommended for patients experiencing severe COVID-19, particularly for those in need of additional oxygen support [25]. However, their administration requires meticulous oversight to prevent the aggravation of hyperglycemia and the risk of adrenal suppression leading to adrenal insufficiency and subsequent hyponatremia [26].

In this review, we discuss the etiology and prognostic value of sodium imbalance in COVID-19 patients, focusing on hyponatremia, in addition to the associated endocrine disruptions caused by SARS-CoV2 infection, more specifically pituitary, adrenal, and thyroid disorders.

## 2. Does the Dysregulation of ACE2 by SARS-CoV-2 Portray a Key Role in COVID-19 Severity?

Examining the renin-aldosterone-angiotensin system (RAAS), a pivotal regulator of blood pressure and lung function accentuates the significance of the angiotensin-converting enzyme (ACE) and the angiotensin-converting enzyme 2 (ACE2). ACE converts angiotensin-I into the vasoconstrictor angiotensin-II (Ang II), while ACE2 foils this mechanism by transforming angiotensin-II into angiotensin 1–7, a vasodilator. The balance between these two pathways is an elemental factor in affecting the appearance of acute and chronic conditions [27].

The initiation of SARS-CoV-2 infection arises when the viral spike (S) protein precisely binds to angiotensin-converting enzyme 2 (ACE2). Contrarily, (ACE) inhibitors and ARBs could potentially serve as novel treatments for patients diagnosed with SARS-CoV-2 [28]. Regardless of the paucity of accessible data, a few observational studies have failed to establish a relation between the seriousness of COVID-19 and the usage of ACE inhibitors and ARBs [29].

The imbalance between ACE and ACE2, induced by SARS-CoV-2, precipitates renin–angiotensin–aldosterone system (RAAS) activation, which ultimately drives the progression of COVID-19. This progression is particularly pronounced in patients with comorbidities such as arterial hypertension, diabetes mellitus, and cardiovascular disease [30]. The increased levels of ACE2 in these patients can be explained by the upregulation of ACE2 as a protective measure to counteract the harmful effects of Ang II [31].

Research has shown that ACE2 receptors are present in the adrenal glands too, particularly in the adrenal cortex, which produces cortisol and aldosterone. This presence suggests that the adrenal glands could be directly affected by SARS-CoV-2, leading to adrenalitis (the inflammation of the adrenal glands), potentially causing adrenal insufficiency [32].

Aldosterone and the natriuretic peptides ANP and BNP function in a counterbalancing manner regarding sodium regulation and fluid equilibrium. Specifically, aldosterone is responsible for enhancing sodium retention and promoting the excretion of potassium, whereas ANP and BNP encourage the excretion of sodium and fluid, thereby contributing to a decrease in blood volume and arterial pressure [33]. Aldosterone, a mineralocorticoid synthesized in the adrenal cortex, operates on the distal tubules and collecting ducts of the kidneys, where it increases sodium reabsorption and facilitates the elimination of potassium. Vasopressin, or ADH, is produced by the posterior pituitary gland and is integral to the regulation of water balance. It enhances water reabsorption in the renal collecting ducts, which subsequently impacts serum sodium concentration. ANP and BNP are peptides secreted by the heart, with ANP derived from the atrial tissue and BNP from the ventricular tissue. These peptides promote the excretion of sodium and water by the kidneys and inhibit the renin–angiotensin–aldosterone system (RAAS), leading to a reduction in both blood volume and blood pressure [34,35,36].

COVID-19 has the potential to disrupt the RAAS, possibly leading to disturbances in sodium concentrations. Such disruptions may manifest as either hyponatremia or hypernatremia, depending on the virus’s influence on the RAAS pathway. By reducing aldosterone levels, sodium loss (hyponatremia) can be induced and contribute to the fluid and electrolyte imbalances observed in COVID-19 patients. In cases of severe COVID-19, changes in aldosterone function can intensify these sodium imbalances, resulting in serious complications such as fluid overload, dehydration, or increased strain on the cardiovascular system, all of which are critical factors in the management of patients affected by COVID-19 [29].

A recently published review examined the viral-induced imbalance between ACE2 substrates and products, contributing to the worsening of COVID-19 severity. By considering the proposed roadmap, multiple therapeutic strategies were suggested to restore balance to the products of ACE2 and improve the symptoms of the disease [37].

## 3. COVID-19 and Endocrine Glands

### 3.1. Endocrine Profile of COVID-19

**Figure 1:**
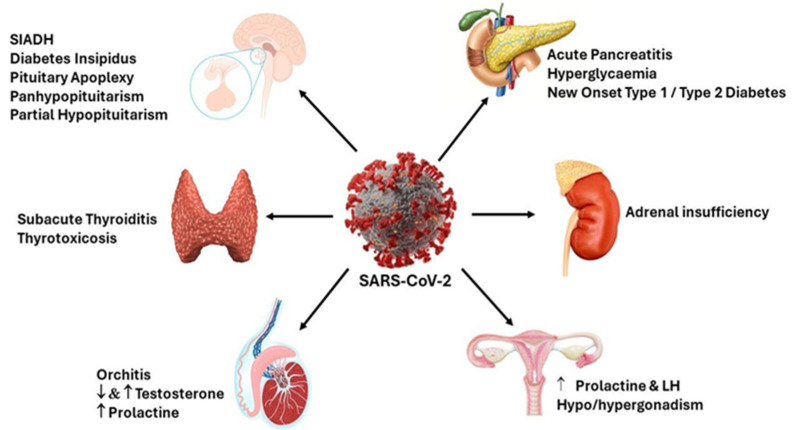
Effects of SARS-CoV-2 on pituitary, thyroid, adrenal, gonadal, and pancreatic endocrine function. SARS-CoV-2—severe acute respiratory syndrome coronavirus 2, SIADH—syndrome of inappropriate antidiuretic hormone secretion, LH—luteinizing hormone, ↓—decreased secretion, ↑—increased secretion.

At first, there were indications that COVID-19 might have an impact on the endocrine system. However, a considerable body of research has since provided detailed insights into the specific effects of the virus on various endocrine glands, including the pituitary, thyroid, adrenal, gonadal, and pancreatic glands [38], shown in Figure 1. These endocrine disorders manifest in a range of endocrine phenotypes, varying from mild symptoms to severe endocrine emergency [39].

Adrenal insufficiency and COVID-19 exhibit a reciprocal influence on one another, with individuals afflicted by adrenal insufficiency face an elevated risk of contracting the infection. This heightened susceptibility is likely due to the decreased production of cortisol in those with adrenal insufficiency [40]. Adrenal insufficiency following acute adrenal infarction and adrenal hemorrhage has been documented in case studies post-COVID-19 [40,41]. SARS-CoV-2 has been shown to inhibit the function of the thyroid gland, leading to conditions like subacute thyroiditis and thyrotoxicosis, as well as affecting the secretion of prolactin and male sex hormones [42]. COVID-19 may adversely affect thyroid function through the direct viral invasion of the thyroid gland. Additionally, the virus can instigate immune system dysregulation, which may aggravate autoimmune thyroid diseases. Disruption of the HPT axis is another consequence, leading to abnormal levels of thyroid hormones. Furthermore, the stress induced by the infection and its treatment may further compromise thyroid function, resulting in both short-term and long-lasting thyroid disorders. Moreover, the virus can target the pancreas, causing acute pancreatitis, hyperglycemia, and the onset of new cases of type 1 or type 2 diabetes [17,43]. COVID-19 can impair both the endocrine function of the pancreas, leading to issues with insulin production and glucose regulation, and its exocrine function, potentially causing pancreatitis and digestive problems [44]. The underlying causes for this phenomenon encompass a multitude of factors, such as stress-induced hyperglycemia, the administration of steroids for COVID-19 treatment, and the direct impact of the virus on pancreatic function [15,26].

The impact of COVID-19 on gonadal function is not extensively documented. Dysfunction of the gonads can result in hypogonadotropic hypogonadism in both sexes and orchitis in males. Among males, the condition commonly presents as orchitis or epididymitis and hypogonadotropic hypogonadism. Some studies have indicated disturbances in spermatogenesis [45,46,47,48]. Conversely, investigations have shown no significant alterations in the ovarian hormonal profile of females affected by SARS-CoV-2 [48].

Nevertheless, a comprehensive interpretation of the extent to which endocrine dysfunction contributes to the symptoms observed in patients with COVID-19 is still debatable.

### 3.2. COVID-19 and the Pituitary Gland

Significant expression of ACE2 mRNA in the hypothalamus and pituitary gland was observed in both animal and human models. In addition, individuals with pituitary dysfunctions, which frequently accompany conditions like obesity, vertebral fractures, and diabetes mellitus, have been found to reveal increased mortality rates and unfavorable outcomes in patients with SARS-CoV-2 infections.

Diverse studies have indicated potential hypothalamo–hypophyseal participation in relation to SARS-CoV-2. These commodiously involve conditions such as hypophysitis, hypopituitarism, pituitary apoplexies, the syndrome of inappropriate antidiuretic hormone secretion (SIADH), and diabetes insipidus. In contrast, examples of hypothalamic involvement have been infrequently documented [49].

Gorbova et al. report on a clinical case that exemplifies the occurrence of reversible hypopituitarism resulting from hypophysitis which developed after a COVID-19 infection [50]. The findings of three separate studies have indicated and highlighted that an isolated pituitary abnormality, specifically in the form of diabetes insipidus, can manifest as a delayed consequence of a COVID-19 infection [51,52,53]. On the other hand, a case study highlighted a male patient who initially presented with symptoms of lower respiratory tract infection, but later developed central diabetes insipidus, only 12 days after being infected with SARS-CoV-2. To conclude, diabetes insipidus should be regarded as a key contender among the top potential differential diagnoses for SARS-CoV-2 patients displaying signs of polyuria and polydipsia [54]. Given its enormous vascular supply and vascular endothelium rich in ACE2 receptors, the pituitary gland is at risk of injury during SARS-CoV-2 infection. Additionally, alteration in platelet function and coagulation amidst COVID-19 infection can lead to pituitary apoplexy [55]. In a recently published prospective study, intriguing discoveries were made regarding the hormonal changes associated with SARS-CoV-2 infection. Although the COVID-19 patients did not exhibit a statistically significant decrease in testosterone levels, there was an observed elevation in serum luteinizing hormone (LH) levels. Furthermore, the ratio of serum testosterone to LH decreased, along with a lower ratio of follicle-stimulating hormone (FSH) to LH, when compared to the control group. These findings might suggest a malfunction of the Leydig cells, which are responsible for testosterone production in the testes, leading to an insufficient production of testosterone in response to SARS-CoV-2 infection [56]. As a result, the hypothalamus compensates for this testosterone decline by increasing the secretion of luteinizing hormone-releasing hormone (LHRH). Consequently, it has been observed that men with critical illness also experience reduced levels of free testosterone [57]. Conflicting results have led to ongoing discussions regarding the role of ovarian function and estradiol in COVID-19 infection and progression. An in-depth analysis of a cohort comprising more than 1000 women who had survived COVID-19 indicated that 46% of them experienced irregular monthly bleeding [58]. Additionally, there were reports of newly emerged dysmenorrhea and changes in menstrual cycle duration [59,60]. COVID-19 and severe illness have an important effect on the hormonal balance in men and women, specifically impacting testosterone and estradiol levels and the functioning of the hypothalamic–pituitary–gonadal axis [61,62,63]. Acute severe illness induces a series of hormonal changes that prioritize the allocation of bodily resources to fight disease, thereby compromising other non-essential functions, such as reproductive capabilities. Research involving both male and female subjects experiencing acute illness or stress has demonstrated decreased levels of circulating sex steroid hormones (estradiol in females and testosterone in males), which correlate with abnormally low gonadotropin levels. This phenomenon indicates a temporary suppression of the hypothalamo–pituitary–gonadal (HPG) axis. Concurrently, there is an activation of the HPA axis, leading to increased serum cortisol levels [47,64].

Women experiencing medical conditions may encounter alterations in their menstrual cycles and fertility, which can be attributed to the underlying disease or the medications they are taking. Typically, these changes are linked to the pathophysiological mechanisms that involve the hypothalamic–pituitary–gonadal–uterine axis. Disruption of this axis can result in anovulation, leading to various menstrual irregularities such as amenorrhea, oligomenorrhea, or sporadic metrorrhagia [48]. These observations accentuate the importance of exploring the association between pituitary dysfunctions and the severity of COVID-19 and the potential implications for clinical management and therapeutic interventions.

### 3.3. COVID-19 and the Adrenal Gland

Adrenal insufficiency (AI) is a clinical condition characterized by the insufficient production of steroid hormones, particularly cortisol, by the adrenal glands. AI is classified into two primary categories: primary and secondary adrenal insufficiency (SAI). Primary adrenal insufficiency (PAI), known as Addison’s disease, is a rare, yet critical disorder, often caused by the autoimmune destruction of the adrenal cortex [65].

In a recent systematic review, Vakhshoori et al. provided a comprehensive analysis of the prevalence of adrenal insufficiency in individuals diagnosed with COVID-19. The findings from various studies included in the review indicated that the occurrence of adrenal insufficiency varied significantly, ranging from 3.1% to 64.3%. These results strongly suggest that adrenal insufficiency is a prevalent condition among patients affected by COVID-19 [66].

The presence of adrenal insufficiency in a study conducted by De Almeida et al. was detected in approximately one-third (33.3%) of patients afflicted with moderate COVID-19. Hyponatremia was present in 75% of patients suffering from adrenal insufficiency, with no cases of hypernatremia reported among the individuals [67]. On the other hand, in a study by Bayaz et al. upon admission on the first day, both deceased and living individuals displayed comparable levels of sodium and potassium electrolytes [68].

Patients with COVID-19 displayed a diminished adrenocortical response, resulting in a considerable number of individuals showing plasma cortisol and ACTH levels that aligned with central adrenal insufficiency [69].

The severity of the disease was found to be positively correlated with cortisol levels, as patients with a cortisol level higher than 744 nmol/L demonstrated a worse prognosis than those with levels below this threshold [70]. Normally, cortisol, as a component of the HPA axis, is generated following a specific diurnal rhythm. This rhythm involves a peak in the morning and a low point at midnight, accompanied by minor pulsatile variations in between. However, the presence of acute illness disrupts this pattern, causing a change in the low point to occur during the night. Increased levels of cortisol in the bloodstream have a significant impact on immune cells through the activation of their receptors [71]. This activation triggers the synthesis of pro-inflammatory cytokines. Consequently, this inflammatory response leads to immune deficiencies and the onset of various metabolic disorders, including type 2 diabetes mellitus, and Addison’s disease [72]. Henceforth, clinicians have the option to evaluate cortisol levels as an additional measure that holds promise in predicting outcomes [73,74].

### 3.4. COVID-19 and the Thyroid Gland

The frequency of thyroid dysfunction in patients with COVID-19 was found to range between 13% and 64% according to a comprehensive analysis of seven studies involving over 1000 individuals. This systematic review provides valuable insights into the prevalence of thyroid dysfunction in COVID-19 patients, highlighting the significant variability observed across different studies [75].

The findings of Muller et al.’s investigation revealed that patients with COVID-19 frequently experience abnormal thyroid function, with the prevailing annotation being a decrease in TSH values. The reduced TSH levels appear to be linked to elevated levels of the inflammatory cytokine [76]. Additionally, the other research indicated a potential correlation between TSH suppression and increased levels of the inflammatory cytokine IL-6 [77].

The results of Yanachkova et al.’s analysis revealed that, following two months post SARS-CoV-2 infection, only 38.9% of the patients remained in a euthyroid state, while the remaining 61.1% displayed abnormal thyroid function test outcomes. Among the various thyroid dysfunctions observed, subclinical hypothyroidism was the most prevalent, affecting 78.3% of the patients. It is worth noting that none of the patients were diagnosed with clinical hyperthyroidism [78].

Gao et al. investigated 100 SARS-CoV-2 patients, the majority of whom (66%) were severely or critically ill. Levels of FT3, TSH, and the FT3/FT4 ratio decreased as patients deteriorated clinically, with non-survivors displaying lower concentrations of these thyroid hormones [79]. A cohort of more than 3500 COVID-19 patients was examined in a retrospective study conducted within the health system of New York City. Among these patients, 6.8% patients had pre-existing hypothyroidism. The study’s findings indicated that there was no significant association between hypothyroidism and an elevated risk of hospitalization, mechanical ventilation, or mortality [80].

The autoimmune/inflammatory syndrome induced by adjuvants (ASIA), also known as Shoenfeld’s syndrome, triggered by adjuvants in certain coronavirus vaccines, has been documented to manifest as thyroiditis and Graves’ disease in vaccinated individuals [81].

These studies have some limitations. Their retrospective natures meant that thyroid function tests were commonly performed only upon admission or after the resolution of the infection, thus preventing the consideration of any dynamic fluctuations in thyroid function during the COVID-19 disease.

Thyroid hormone concentrations can oscillate in reaction to acute illnesses, a condition referred to as non-thyroidal illness syndrome (NTIS) or euthyroid sick syndrome [82]. This condition is characterized by irregular thyroid hormone levels that arise without any underlying dysfunction of the thyroid gland itself. In NTIS, the pre-dominant hormonal pattern is marked by reduced levels of T3, while T4 may present as either normal or low, and TSH levels can exhibit variability, being low, normal, or slightly elevated. This syndrome is hypothesized to be a consequence of alterations in the metabolism of peripheral thyroid hormones, disruptions in the regulatory functions of the HPT axis, and the reduced conversion of T4 to T3, which is influenced by many cytokines and various mediators of the disease [83]. It is crucial to distinguish NTIS from primary thyroid disorders, including hypothyroidism and hyperthyroidism. NTIS is generally a temporary condition that tends to resolve concurrently with the improvement of the underlying cause [84].

To conclude, patients with uncontrolled thyroid dysfunction may be more prone to complications from infections, as thyroid hormones play a significant role in the immune system [85].

### 3.5. Vaccination against SARS-CoV-2 and Hypopituitarism

The main question is whether vaccination could have a role in the deficits detected in the ante hypophyseal region. The most prevalent manifestations consist of hypophysitis accompanied by ADH deficiency, PAI, and the SIADH. These symptoms typically become apparent shortly after the vaccine has been administered [86].

In one case report, a patient was diagnosed with hypophysitis two days following the administration of the second Moderna vaccine dose. The patient exhibited symptoms of secondary adrenal insufficiency (hyponatremia), central hypothyroidism, and central hypogonadism. Treatment with steroids and L-thyroxine replacement therapy proved beneficial for the patient [55].

Molecular mimicry, vaccine adjuvants, and vaccine-induced thrombotic thrombocytopenia (VITT) are among the potential pathogenetic mechanisms, wherein the involvement of ACE2 receptors in the hypothalamus–pituitary system is significant. The etiology of hypophysitis may also be autoimmune, considering the occurrence of various autoimmune conditions following SARS-CoV-2 vaccination [87].

The safety data concerning COVID-19 vaccines provide reassurance, leaving no grounds to dispute the importance of this immunization in effectively reducing the severity of COVID-19 infections [88].

### 3.6. Pituitary Gland and Post COVID-19 Syndrome

Sequelae resulting from COVID-19 infection can persevere beyond the initial phase of illness, displaying the establishment of a unique medical entity known as Post COVID-19 Syndrome. These sequelae bear resemblance to deficiencies in anterior pituitary hormones, specifically corticotropin and somatotropin deficiencies, indicating the potential involvement of the hypothalamic–pituitary axis in the manifestation of prolonged symptoms associated with COVID-19 [89].

A recent study by Uhran et al. examined the anterior pituitary axes of patients post COVID-19 recovery, three months later. The results demonstrated that 46.5% of the patients displayed somatotropic deficits, 16.2% exhibited corticotrophic deficits, 9.3% had hypogonadism, and 4.6% had hyperprolactinemia [90].

In a prospective, observational study that was conducted by Rashmi et al., a total of 13.63% of patients exhibited disturbances in the HPA axis following a minimum of 3 months of recovery from COVID-19 infection. The transient nature of adrenal insufficiency with COVID-19 suggests that spontaneous recovery is expected [91].

## 4. Electrolyte Imbalances during COVID-19

### 4.1. Underlying Pathophysiological Mechanisms of COVID-19 Induced Dysnatremia

Dysnatremia, also referred to as sodium imbalance, is a typically observed electrolyte variability in hospitalized individuals. This state encompasses both hyponatremia and hypernatremia, which are variations in the plasma sodium concentration [92].

From a pathophysiological perspective, hyponatremias can be categorized into two distinct groups: hyponatremia caused by the non-osmotic hypersecretion of vasopressin, which can be additionally classified as hypovolemic, hypervolemic, or euvolemic, and hyponatremia originating from non-hypervasopressinemic factors, such as pseudohyponatremia, water intoxication, or cerebral salt-wasting syndrome. European guidelines designate hyponatremia biochemically as mild (pNa 130–135 mmol/L), moderate (125–129 mmol/L), or profound (<125 mmol/L) [93]. The most preeminent, severe hyponatremia, is established on the existence of symptoms or signs of cerebral edema, increased intracranial pressure, a decreased level of consciousness, and finally, yet importantly, a coma [94].

We systematically searched PubMed. This database was searched up to 20 May 2024, using the following keywords: ‘SARS-CoV-2′, ‘COVID-19′, and ‘dysnatremia’, shown in Table 1.

The pooled analysis of Berni et al. showed that hyponatremia was described in nearly 30–60% of SARS-CoV-1-infected patients [104]. According to the study of Ruiz-Sanchez et al., hyponatremia existed in 18.4% of patients with COVID-19, and hypernatremia existed in 2.2% [103]. As mentioned in Frontera et al.’s analysis, hyponatremia appeared in almost a third of SARS-CoV-2-hospitalized patients [105]. A large meta-analysis of 23 studies, conducted by Khidir et al. showed that hyponatremia among COVID-19 patients is associated with increased odds of mortality, an intensive care unit (ICU) stay, and the length of stay [106].

Dysnatremia is sporadically also observed in patients with mild COVID-19, and when it does occur, it is typically of a mild and transient nature. Components such as dehydration, pyrexia, and moderate systemic inflammation may play a role in its development. Direct investigations into the association between mild COVID-19 not requiring hospitalization and dysnatremia are relatively limited; still, the current literature implies that dysnatremia can arise in mild cases of COVID-19, though it is observed less frequently than in severe cases. However, some studies and reviews provide insight into the occurrence of dysnatremia in the broader context of COVID-19, including in patients with milder forms of the disease [96,107].

As already mentioned, SARS-CoV has been linked to a range of endocrine disorders, with a specific focus on those related to the pituitary gland. Leow et al., in 2005, was the first to present documentation of abated pituitary function in individuals with coronaviruses, more precisely SARS-CoV-1 [108]. The SIADH is the main culprit beyond hypotonic hyponatremia, promoted by the nonosmotic deliverance of arginine vasopressin (AVP, previously recognized as an antidiuretic hormone), which interacts with the renal V2 receptors to stimulate water conservation [109].

**Figure 2:**
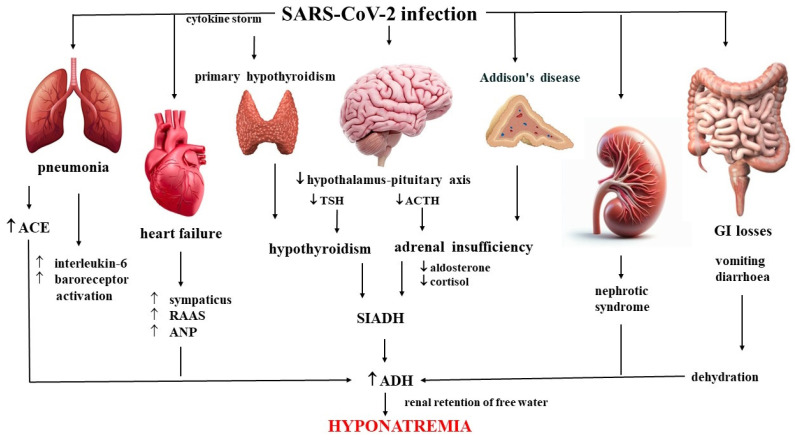
The pathophysiological mechanisms of SARS-CoV-2 induced hyponatremia. ACE—angiotensin-converting enzyme, ACTH—adrenocorticotropic hormone, ADH—antidiuretic hormone, ANP—atrial natriuretic peptide, GI—gastrointestinal. SIADH—syndrome of inappropriate antidiuretic hormone secretion, ↓—decreased secretion, ↑—increased secretion.

The development of hyponatremia can be explained by several plausible mechanisms, as shown in Figure 2. Firstly, COVID-19 can trigger the SIADH, a condition characterized by the excessive release of antidiuretic hormone (ADH) despite normal or low plasma osmolality. Increased ADH levels lead to water retention, diluting the sodium concentration in the blood and resulting in hyponatremia [110].

The SIADH in COVID-19 may be induced by inflammation, stress, and lung involvement (such as pneumonia or acute respiratory distress syndrome), which are known triggers for ADH release. Severe lung involvement in COVID-19 can stimulate pulmonary stretch receptors and baroreceptors, leading to non-osmotic ADH release [111]. This response, meant to preserve blood pressure during respiratory distress, can contribute to water retention and hyponatremia [112]. SARS-CoV-2 has been associated with a significant overproduction of inflammatory cytokines, which can potentially instigate the occurrence of a cytokine storm [113]. This surge in cytokines can contribute to the development of SIADH through two separate mechanisms. Firstly, specific inflammatory cytokines like IL-6 can directly stimulate the release of ADH without any alteration in osmolarity. Secondly, these cytokines can cause damage to the lung tissue and alveolar cells, triggering SIADH through the hypoxic pulmonary vasoconstriction pathway. Interleukin 6 (IL-6), a significant cytokine, plays a crucial role in the pathogenesis of COVID-19 [114]. Atila et al., in 2022, reported that a more notable inverse correlation between IL-6 concentration and plasma sodium levels was observed in COVID-19 when compared to other bacterial and viral infections [115]. Cytokines are crucial in modulating the HPA. Pro-inflammatory cytokines, such as interleukin-1 (IL-1), IL-6, and tumor necrosis factor-alpha (TNF-α), activate the HPA axis by targeting the hypothalamus, which triggers the secretion of corticotropin-releasing hormone (CRH). This hormone then prompts the pituitary gland to release adrenocorticotropic hormone (ACTH), leading to cortisol production by the adrenal glands. Cortisol, the final product of HPA axis activation, exerts a feedback effect on both the hypothalamus and the pituitary gland, inhibiting further CRH and ACTH release. The influence of cytokines on this feedback loop can vary, either amplifying or reducing it based on the specific context and type of cytokine involved. Chronic inflammation may result in sustained HPA axis activation, potentially causing imbalances in cortisol levels and contributing to stress-related disorders or immune dysregulation. The interplay between cytokines and the HPA axis is intricate, reflecting a dynamic equilibrium between immune responses and hormonal control [49,116,117].

COVID-19 can cause adrenal insufficiency or secondary hypocortisolism due to the stress-induced suppression of the hypothalamic–pituitary–adrenal (HPA) axis or direct viral effects on the adrenal glands or the pituitary gland. Reduced cortisol levels impair the kidneys’ ability to excrete free water and may increase ADH secretion, both of which contribute to hyponatremia. The specific percentages observed can differ depending on the severity of the condition, the presence of comorbidities, and the context of healthcare delivery [118]. In a study by Bayaz et al. involving patients with normal adrenal function, 22.5% exhibited hyponatremia, while another 22.5% demonstrated hypernatremia. In stark contrast, 75% of individuals with adrenal insufficiency were found to have hyponatremia, with no cases of hypernatremia reported. Notably, there was no significant difference in potassium levels between the two patient groups [68].

Thyroid function can be disrupted by the SARS-CoV-2 virus, leading to hypothyroidism. This condition is associated with reduced renal perfusion and a reduced glomerular filtration rate (GFR), which can enhance water retention and increase ADH activity. Furthermore, the combination of reduced renal function and increased ADH can lead to dilutional hyponatremia in hypothyroid patients [119].

The virus also binds to ACE2 receptors, which are critical for regulating the RAAS. Disruption of ACE2 can lead to reduced aldosterone production, impairing sodium reabsorption in the kidneys and contributing to sodium loss. Additionally, the imbalance in the RAAS can lead to fluid retention and the further dilution of sodium levels [120].

In addition, dehydration induced by gastrointestinal losses such as diarrhea and vomiting leads to the direct loss of sodium and other electrolytes, which can contribute to hyponatremia [121]. On the other hand, heart failure and nephrotic syndrome cause a reduced effective circulatory volume, leading to an increased effect of ADH [122].

### 4.2. Hypernatremia and COVID-19

The classification of hypernatremia into mild, moderate, or severe is determined by the plasma sodium concentration levels surpassing 145, 150, and 155 mmol/L, respectively. Various pathophysiological mechanisms can be attributed to hypernatremia in patients with COVID-19, with the gastrointestinal tract and kidneys playing a key role in disrupting the delicate balance of water and electrolytes [123].

The lack of association between plasma sodium concentration and sodium input, the persistent occurrence of hypernatremia despite targeted therapeutic measures, and the established binding of SARS-CoV-2 to the ACE2 receptor collectively indicate an abnormal elevation in renal sodium reabsorption due to heightened angiotensin II activity secondary to SARS-CoV-2 infection [124,125].

The prevalence of hypernatremia in patients undergoing treatment in medical intensive care units (ICUs) displayed considerable variability, with percentages ranging from 6% to 26% [126]. A large meta-analysis revealed a notable contrast in the mortality rates between patients with COVID-19 in the normonatremia group (18%) and the hypernatremia group (48%). The proportion of deaths in the normonatremia group was significantly lower than that in the hypernatremia group [123].

In the future, it is important for physicians to expeditiously recognize the risk factors linked to hypernatremia in their clinical practice. Furthermore, patients who are already experiencing hypernatremia upon admission should be carefully monitored and provided with adequate treatment to mitigate the risk of hypernatremia and decrease mortality rates in individuals with COVID-19 [127].

### 4.3. Dysnatremia as a Prognostic Factor for COVID-19 Mortality

Hypernatremia demonstrates a specificity of 97% in predicting a negative prognosis, with the correlation being unaffected by the levels of inflammatory markers [128].

Higher mortality rates have been observed in patients admitted to the hospital with dysnatremia, as opposed to those with normonatremia. In particular, hyponatremia has been associated with the progression of severe disease and an increased likelihood of death. This is likely due to its correlation with other severe conditions, such as acute respiratory distress syndrome (ARDS) and multiorgan failure, which are common complications in severe cases of COVID-19 [95,129,130].

The incidence of hyponatremia varies significantly across different populations and clinical environments. In hospitalized patients, the prevalence is reported to be between 15% and 30% [131]. For critically ill patients, especially those in intensive care units (ICUs), this figure can rise to between 30% and 50% [132]. The elevated prevalence in this cohort is largely due to the severity of their conditions, the impact of pharmacological treatments, and existing health issues that influence sodium regulation. Among older adults, hyponatremia is also prevalent, with reported rates ranging from 10% to 40% [124]. This increased occurrence can be linked to factors such as the use of multiple medications, reduced kidney function, and various comorbidities. In patients diagnosed with heart failure, the prevalence of hyponatremia is observed to be between 20% and 30%, while in those with liver cirrhosis, it is found in approximately 30% to 50% of cases [133,134]. Additionally, individuals with pneumonia frequently experience hyponatremia, with prevalence rates estimated at 10% to 30% [135,136].

In the end, dysnatremia, a well-known prognostic factor in all hospitalized patients could also be a crucial prognostic component in determining the mortality rate of COVID-19. This contributes to the existing set of clinical parameters employed to anticipate patient outcomes and inform treatment strategies.

## 5. Future Directions and Conclusions

Future studies are needed to elucidate the precise mechanisms by which SARS-CoV-2 affects sodium balance and hormone secretion. Understanding the pathways through which the virus influences the hypothalamic–pituitary–adrenal (HPA) axis, thyroid and adrenal function, as well as the hypothalamic regulation of thirst and fluid balance can provide insights into the development of hyponatremia in COVID-19 patients. Moreover, investigating the interactions between inflammatory cytokines and endocrine function could enhance our understanding of the SIADH during COVID 19. In addition, longitudinal studies are essential to assess the persisting effects of infection on endocrine function. Research focusing on thyroid dysfunction and adrenal insufficiency in the post-COVID period will be crucial to determine the long-lasting effects on hormone levels and overall health outcomes. Furthermore, identifying specific biomarkers such as inflammatory markers, electrolyte levels, and hormonal assays that correlate with disease severity and treatment response could help in developing targeted interventions for patients at risk. Investigating the impact of pre-existing endocrine disorders on COVID-19 outcomes is yet another critical research direction. Studies should focus on how conditions such as adrenal insufficiency, hypothyroidism, and hyperthyroidism affect the susceptibility to severe COVID-19, the incidence of hyponatremia, and overall outcomes. Such research could help develop tailored strategies for managing at-risk populations. Exploring whether vaccinated individuals experience different endocrine response profiles compared to unvaccinated individuals, especially in terms of hormonal balance and electrolyte homeostasis after breakthrough infections, could also be of great value.

COVID-19 represents a complex challenge to global health, with its effects extending far beyond respiratory symptoms. The interplay between the virus and various endocrine disturbances, particularly hyponatremia, the SIADH, thyroid dysfunction, and adrenal insufficiency, underscores the need for a comprehensive approach to patient management. As research continues to evolve, understanding these multifaceted interactions will be vital for developing effective treatment strategies and improving patient outcomes during and after the pandemic. Therefore, understanding dysnatremia as well as the role of hormonal dysregulation in COVID-19 is essential for optimizing patient care, guiding treatment strategies, and improving clinical outcomes in affected individuals.

## Figures and Tables

**Table 1:** Studies that have described dysnatremia in COVID-19 patients—a summary of key studies.

Authors	Type of Study	Country	Year of Publication	Number of Patients	Hyponatremia on Admission/ Fatal Outcome	Hypernatremia on Admission/ Fatal Outcome
Khan et al. [95]	Retrospective longitudinal study	Pakistan	2023	574	39%	4.7%
Liu et al. [96]	Retrospective observational study	USA	2022	5407	7%/8%	7%/15%
Królicka et al. [97]	Retrospective observational study	Poland	2023	2026	17.14%/28.52%	5.03%/47.95%
Núñez-Martínez et al. [98]	Retrospective, descriptive and analytical cohort study	Mexico	2022	722	21.19%/13.3%	2.49%/13.7%
Martino et al. [99]	Open-label, observational study	Italy	2021	117	26.5%/4%	6.8%/1%
Voets et al. [100]	Retrospective chart review	USA	2021	331	34%	38%
Tzolius et al. [101]	Retrospective longitudinal cohort study	United Kingdom	2021	488	24.6%/28.4%	5.3%/46.1%
Atila et al. [102]	Prospective, observational, cohort study	Switzerland	2021	1041	28.1%/11.5%	2.9%/9.3%
Ruiz-Sánchez et al. [103]	Retrospective study	Canada, Germany, China, Cuba, Italy, Spain, Ecuador	2020	5868	18.4%	2.2%

## Data Availability

The original contributions presented in the study are included in the article, further inquiries can be directed to the corresponding author.
